# Psychological distress among medical students in conflicts: a cross-sectional study from Syria

**DOI:** 10.1186/s12909-017-1012-2

**Published:** 2017-09-20

**Authors:** Tareq Al Saadi, Sarah Zaher Addeen, Tarek Turk, Fatima Abbas, Mahmoud Alkhatib

**Affiliations:** Faculty of Medicine, University of Damascus, Damascus, Syrian Arab Republic

**Keywords:** Medical students, Depression, Anxiety, Stress, Syrian war, Syria

## Abstract

**Background:**

Medical education can be a time of great psychological distress for students. The ongoing Syrian conflict represents an additional factor potentially contributing to poor mental health among medical students. Studies revealed high levels of psychological distress among Syrians. We aimed to investigate the prevalence and risk factors of depression, anxiety and stress among medical students at Damascus University during this period of war.

**Methods:**

A cross-sectional study was conducted using the Depression, Anxiety and Stress Scale (DASS-21) in addition to questions about demographic and financial characteristics, and questions about the effects of the ongoing war on the participants’ lives.

**Results:**

350 students were included. Prevalence of depression, anxiety and stress was 60.6%, 35.1%, and 52.6%, respectively. Depression was more likely in females and those with “intermediate” or “insufficient” personal income. Anxiety was more likely in females and those with “insufficient” personal income while less likely in fifth- and sixth-year compared to second-year students. Stress was lower in fifth-year compared to second-year students and higher in “insufficient” personal income compared to “sufficient” personal income.

**Conclusions:**

We concluded that Syrian medical students suffer from high rates of psychological distress. Females, second-year students, and those with “insufficient” personal income were the most affected. Students’ perception of their own financial status, rather than the financial status per se was related to psychological distress. There was no evidence of a direct relationship between the ongoing conflict and psychological distress. Further investigations of causes and consequences of poor mental health in Syrian medical students are essential.

**Electronic supplementary material:**

The online version of this article (10.1186/s12909-017-1012-2) contains supplementary material, which is available to authorized users.

## Background

Medical education aims to produce well-trained physicians who are capable of taking the responsibility of advancing public health and achieving high levels of patient-centered care. This requires many years of stressful studying and persistent training. The constant struggle that students go through to become highly qualified healthcare providers might, in some cases, result in psychological distress [1, 2]. In addition, students’ mental and emotional health can be unintentionally negatively affected by some aspects of training [3]. Factors that might contribute to such psychological problems among medical students include exposure to patients’ sufferings [4], financial matters [5], lack of sleep [5], and student abuse (verbal, psychological or physical abuse in a medical setting) [6].

Depression is the leading cause of disability worldwide [7]. It involves a variety of symptoms including depressed mood, loss of interest and enjoyment, reduced energy, disturbed sleep and appetite, feelings of guilt or low self-worth, and poor concentration [7]. Anxiety is an emotion that is accompanied by tension, worried thoughts and subsequent physical changes, like elevated blood pressure [8]. Stress becomes abnormal when it interferes with the normal life, causing fatigue, inability to concentrate, or irritability [9].

Many studies have documented the negative side effects of war on mental health [10]. Started in 2011, the Syrian war keeps exposing civilians to physical and psychological trauma on a daily basis. The ongoing conflict represents an additional factor potentially contributing to mental distress among medical students in Syria. As there is a lack in published studies about the mental health profiles in Syria [11], and because the mental health of the Syrian medical students has not been investigated before, we conducted this study to investigate the prevalence and the risk factors of depression, anxiety and stress among medical students at Damascus University during this period of war. To our knowledge, this is also the first study to investigate psychological distress among medical students in the setting of conflicts.

## Methods

### Study design

A cross-sectional study regarding the prevalence and risk factors of psychological distress among medical students was conducted in November 2015.

### Participants

All participants were current medical students at Damascus University. Participants were recruited from second- through sixth-year of the 6-years program of Faculty of Medicine of Damascus University. First-year students were excluded from our study because this year is considered a pre-medical preparatory year and a substitute for the first year in faculties of Medicine, Dentistry and Pharmacy.

### Data collection

An anonymous online questionnaire was designed using Google Forms, and was distributed to medical students in Faculty of Medicine of Damascus University via students’ online platforms.

Convenience sampling was used to recruit participants, and participation was available for all students from second through sixth year. The objectives of the study were explained to the participants who were informed that their participation was voluntary, and anonymity was assured. The participants were also told that the results of this research will be published. Filling out the questionnaire and submission by the student himself/herself was considered as a declaration of willingness to participate.

### The questionnaire

The Depression, Anxiety, and Stress Scale (DASS-21) [12] was used to assess the psychological distress among participants by investigating the symptoms of depression, anxiety, and stress. It is a 21-item questionnaire with a four-point (0–3) answer scale. The questions ask about the extent participants had experienced certain symptoms over the previous week. These items are also arranged into subscales; depression, anxiety and stress; 7 items for each subscale. Each subclass’s score equals the sum of seven corresponding questions. The sum scores are multiplied by 2 in order to match the original scale score in DASS-42. Each subscale score ranges from 0 to 42 [12]. For depression, scores less than 9 are considered ‘normal’, 10–13 are ‘mild’, 14–20 are ‘moderate’, 21–27 are ‘severe’ and scores greater than 28 are considered ‘extremely severe’. Scores less than 7 on the anxiety subscale meet ‘normal’ category, while 8–9 are considered ‘mild’, 10–14 are ‘moderate’, 15–19 are ‘severe’ and scores above 20 are considered ‘extremely severe’. Stress scores below 14 meet ‘normal’ status, scores between 15 and 18 meet ‘mild’ stress, 19–25 correlate with ‘moderate’ stress, 26–33 are ‘severe’ stress and scores greater than 34 meet ‘extremely severe’ stress [12].

DASS is a reliable tool to assess psychological distress in clinical and non-clinical populations [12, 13], Together, the subscales provide a broad-spectrum measure of psychological distress, indicating the severity and frequency of symptoms [14]. DASS-21 requires less time to administer, and it is superior to the full-scale version [14]. We administered the validated Arabic version of DASS-21 [15].

In addition, we asked the participants to complete a specially designed questionnaire that aimed to investigate the factors associated with psychological distress. This survey contained questions about demographic characteristics (gender, year of study, nationality, marital status, residence state), financial characteristics (monthly family income, personal income), and habits (cigarettes and water pipe smoking status), as well as questions about the effects of the ongoing war on their lives (residence change due to war, physical or financial damage to self or first-degree family member due to war). This survey was developed for this study by the authors and an English version of it is presented as Additional file 1.

### Statistical analysis

Participants’ characteristics were reported as frequencies and percentages.

Subscale scores for depression, anxiety and stress were summed as per the DASS manual, and were multiplied by 2 because we had used the DASS-21 version of the scale. The scores were reported as means and standard deviations. DASS scores were then categorized as ‘normal’, ‘mild’, ‘moderate’, ‘severe’ and ‘extremely severe’ as per the DASS manual [12]. The proportion of participants with scores in the normal and mild range were classified as “psychologically normal”, while those with scores in the moderate to extremely severe range were classified as “psychologically distressed”. Distribution of participants on these categories was reported as frequencies and percentages.

For each type of psychological distress, a binary logistic regression analysis was calculated to predict participants’ probabilities of being psychologically distressed based on their characteristics. The dependent variable was the presence of the particular psychological distress, and the independent variables included all the investigated characteristics of the participants. The backward conditional method was applied to produce a final model that only contains the statistically significant variables affecting the particular distress. Results of logistic regression were reported as adjusted odds ratio (AORs) with 95% confidence intervals (CIs).

All statistical analyses were carried out with the Statistical Package for Social Sciences version 22.0 (SPSS Inc., Chicago, IL, United States). A *p*-value of less than 0.05 was the level of significance in all statistics.

## Results

### Participants’ characteristics

Overall, 350 students completed the questionnaire and provided their consent to participate in the study. Table 1 describes the characteristics of the study sample. The sample contained 148 males (42.3%) and 202 females (57.7%). Current year of study for participants ranged from second- through sixth-year. The majority of the participants were Syrians (91.4%), singles (90.6%), living at home with family or relatives (73.1%), and non-smokers (81.1%). About half of the participants reported that they had changed their residence due to the ongoing war in Syria (49.1%), and that themselves, or a first-degree family member, had suffered from physical or financial damage due to the war (51.1%). Responses for monthly family income and the perspective about personal income varied widely between the participants (Table 1).

**Table 1:** Demographic characteristics of participants (*N* = 350)

Characteristic	N (%)
Gender:	
Male	148 (42.3%)
Female	202 (57.7%)
Year of study:	
Second	85 (24.3%)
Third	41 (11.7%)
Fourth	97 (27.7%)
Fifth	92 (26.3%)
Sixth	35 (10.0%)
Nationality:	
Syrian	320 (91.4%)
Palestinian	24 (6.9%)
Other	6 (1.7%)
Marital Status:	
Single	317 (90.6%)
In a relationship/Engaged	31 (8.9%)
Married	1 (0.3%)
Separated/Divorced/Widowed	1 (0.3%)
Residence Status:	
Home - with family or relatives	256 (73.1%)
Home - alone	12 (3.4%)
Home - with other students	29 (8.3%)
On-campus housing	53 (15.1%)
Residence changed due to war:	
No	178 (50.9%)
Yes	172 (49.1%)
Physical or financial damage to self or 1st degree family member due to war:	
No	171 (48.9%)
Yes	179 (51.1%)
Monthly family income:	
Less than 30 K S.P.	44 (12.6%)
30 K to 60 K S.P.	114 (32.6%)
60 K to 90 K S.P.	99 (28.3%)
More than 90 K S.P.	93 (26.6%)
Personal income:	
Sufficient	136 (38.9%)
Intermediate	143 (40.9%)
Not sufficient	71 (20.3%)
Cigarettes and waterpipe smoking status:	
Not smoker	284 (81.1%)
Current smoker	58 (16.6%)
Former smoker	8 (2.3%)

*Abbreviations*: *S.P.* Syrian Pounds

### Prevalence of psychological distress

**Fig. 1:**
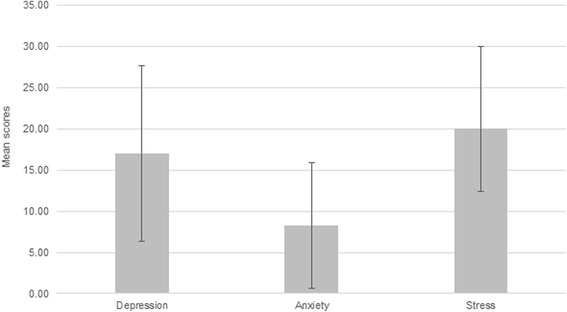
Means and standard deviations of the participants’ DASS-21 subscales scores

The overall mean of the depression subscale scores was 17.01 and the standard deviation was 10.62. Stress subscale scores had a similar distribution with a mean of 20.00 and a standard deviation of 9.97. Scores of these two subscales ranged from 0 to 42. Anxiety subscale scores had lower values of mean and standard deviation (8.31 and 7.63, respectively) and ranged from 0 to 38. The overall means and standard deviations of the DASS-21 subscale scores are represented in Fig. 1.

Distribution of participants based on the categorization of their DASS-21 subscale scores is shown in Table 2. Of the studied psychological distress types, depression was the most prevalent among participants with 60.6% of them having moderate, severe, or extremely severe depressive symptoms. Stress was the second most common psychological distress with a prevalence of 52.6%, while only about one-third (35.1%) of the participants suffered from anxiety.

**Table 2:** Prevalence of psychological distress and distribution of participants based on the categorization of their DASS-21 subscale scores according to DASS manual

	Depression N (%)	Anxiety N (%)	Stress N (%)
Psychologically normal	138 (39.4%)	227 (64.9%)	166 (47.4%)
Normal	95 (27.1%)	190 (54.3%)	118 (33.7%)
Mild	43 (12.3%)	37 (10.6%)	48 (13.7%)
Psychologically distressed	212 (60.6%)	123 (35.1%)	184 (52.6%)
Moderate	102 (29.1%)	71 (20.3%)	78 (22.3%)
Severe	41 (11.7%)	20 (5.7%)	63 (18.0%)
Extremely severe	69 (19.7%)	32 (9.1%)	43 (12.3%)

### Predicting factors for psychological distress

Final models of binary logistic regression for depression, anxiety, and stress contained only the significant predicting variables (Table 3).

**Table 3:** Factors associated with depression, anxiety, and stress symptoms among participants

	*P*-value	AOR	95% C.I. for AOR	
			Lower	Upper
Depression**:** (χ^2^ = 23.526, df = 3, *p*-value <0.001, Nagelkerke R^2^ = 0.088)				
Gender				
Male (ref.)				
Female	0.004	1.926	1.227	3.024
Personal income	0.000			
Sufficient (ref.)				
Intermediate	0.020	1.779	1.096	2.889
Not sufficient	0.000	3.603	1.875	6.923
Constant	0.061	0.662		
Anxiety: (χ^2^ = 23.487, df = 7, *p*-value = 0.001, Nagelkerke R^2^ = 0.089)				
Gender				
Male (ref.)				
Female	0.004	2.036	1.262	3.282
Year of study	0.047			
Second (ref.)				
Third	0.479	0.756	0.348	1.641
Fourth	0.098	0.593	0.319	1.101
Fifth	0.005	0.395	0.208	0.753
Sixth	0.046	0.406	0.167	0.985
Personal income	0.034			
Sufficient (ref.)				
Intermediate	0.328	1.295	0.771	2.175
Not sufficient	0.009	2.292	1.226	4.284
Constant	0.007	0.436		
Stress: (χ^2^ = 15.430, df = 6, *p*-value = 0.017, Nagelkerke R^2^ = 0.058)				
Year of study	0.084			
Second (ref.)				
Third	0.668	0.846	0.393	1.819
Fourth	0.634	0.864	0.473	1.577
Fifth	0.013	0.465	0.254	0.853
Sixth	0.119	0.524	0.233	1.180
Personal income	0.033			
Sufficient (ref.)				
Intermediate	0.055	1.601	0.991	2.587
Not sufficient	0.016	2.081	1.144	3.786
Constant	0.728	1.095		

*Abbreviations*: *AOR* adjusted odds ratio, *CI* confidence interval, *ref.* reference group, *χ* ^*2*^ Chi-square

Gender and personal income were the only significant factors that predicted the presence of depression in the study sample. Females compared to males were about 2 times more likely to be depressed (AOR = 1.926; 95% CI 1.227, 3.024). In addition, those who reported their personal income to be “intermediate” or “not sufficient” were significantly more likely to be depressed (AOR = 1.779; 95% CI 1.096, 2.889 and AOR = 3.603; 95% CI 1.875, 6.923, respectively) than those who reported having a “sufficient” personal income.

For anxiety, the only significant factors were gender, year of study, and personal income. The odds of anxiety were significantly higher in females (AOR = 2.036; 95% CI 1.262, 3.282) compared to males, and in a “not sufficient” personal income (AOR = 2.292; 95% CI 1.226, 4.284) compared to a “sufficient” personal income. On the other hand, being enrolled in the fifth or sixth year was associated with a significantly lower likelihood of having anxiety (AOR = 0.395; 95% CI 0.208, 0.753 and AOR = 0.406; 95% CI 0.167, 0.985, respectively) compared to being enrolled in the second year.

In the final model of stress, both year of study and personal income showed significant associations. Fifth-year students were significantly less likely to have stress than second-year students (AOR = 0.465; 95% CI 0.254, 0.853), and those with “not sufficient” personal income were significantly more likely to be stressed (AOR = 2.081; 95% CI 1.144, 3.786) compared to those who consider their personal income “sufficient”.

## Discussion

Medical education is associated with a great psychological distress that affects many aspects of students’ lives, including their academic performance and professional development [3, 16]. However, the role of demographic and personal variables in this high prevalence of distress is not well understood [16].

### Prevalence

The data reveal high levels of psychological distress. Depression was the most prevalent distress with a prevalence of 60.6%, while approximately one in every two medical students was stressed (52.6%), and one in every three medical students suffered from symptoms of anxiety (35.1%).

Similar studies in developing countries yielded lower prevalence of depression and stress (27.63% for depression in Lebanon [17], 30%–43% for depression and 30%–41% for stress in Saudi Arabia [18], 35.1% for depression in Pakistan [19] and 38.2% for depression in Brazil [20]), and higher prevalence of anxiety (69% in Lebanon [17], 47%–63% in Saudi Arabia [18] and 47.7% in Pakistan [19]). On the other hand, similar studies conducted in more developed countries yielded lower levels of depression (49% in the US [21], 16.4% for moderate to severe depression in the UK [22], and 12.9% in Sweden [23]). However, having used different scales for detecting symptoms of depression and anxiety, comparing the results of these studies to ours may be done with caution.

In their systematic review, Dyrbye et al. [16] found that in 5 studies comparing mental health between medical students and general population, the overall psychological distress was greater among medical students. Furthermore, in a study by Dahlin et al. [23], medical students had significantly higher prevalence of depressive symptoms compared to general population. However, for Syria, more studies about the prevalence of psychological distress in general population are needed in order to make that comparison.

### Effects of demographics and habits

Female gender was shown to be associated with higher levels of depression, anxiety, and stress [16, 18, 19, 23, 24]. In our study, females were approximately two times more likely to be depressed and anxious than males. However, the two genders were not significantly different regarding stress.

The academic year of study was a significant factor in predicting the presence of anxiety and stress, but not depression. Medical students from third through sixth year had lower probabilities of being anxious or stressed than second-year students. However, these differences were significant only for fifth- and sixth-year students in anxiety and fifth-year students in stress. Our findings line with the findings of other studies that suggest the early years of medical schools to be the most stressful [19, 23]. It was found that first-year medical students reported the highest degree of being overwhelmed by the curriculum [23]. Students of more advanced years might have developed abilities to handle the burdens of studying medicine and to adapt with the huge workload, reducing the subsequent stress and anxiety. The gradual shift in the orientation of subjects from being basic sciences-oriented in the early years of medical school to being more clinically-oriented in the advanced years could have affected the distribution of anxiety and stress in our study sample. Other studies, however, revealed a different distribution for psychological distress regarding the year of study [18, 24]. In addition, although some studies found a significant relationship between year of study and depression [18, 20], we did not have a similar relationship in our study. The differences in curricula, teaching and assessment methods between countries may explain these variations.

Nationality, marital status, residence state, and smoking status did not appear to significantly affect the probability of being psychologically distressed. However, our sample had an unbalanced distribution regarding those four aspects (Table 1) which may prevent drawing credible conclusions.

### Effects of finance

The ongoing conflict in Syria had an enormous impact upon economics [25]. Reports estimated that four in every five Syrians lived in poverty during 2014 [25].

As poverty has been consistently linked to poor mental health [26], we investigated the financial properties of the participants as potential factors negatively affecting mental health. Because there are only minimal fees for studying at the Damascus University, the enrolled students vary widely in their socioeconomic backgrounds. We asked the participants to provide inputs about two aspects: monthly family income (as most students in Syria are financially supported by their families) and their own opinions about their income. We found that the monthly family income did not have a significant effect on psychological distress among medical students, unlike the students’ attitude towards their personal income, however, which was a significant factor in predicting depression, anxiety, and stress. In general, psychological distress was significantly higher in those who reported having a “not sufficient” personal income.

While some studies in the literature found income to be a significant factor negatively affecting psychological health [27], others did not show a similar relationship [19, 24, 28]. Ross et al. noted a possible negative effect for worrying about money upon performance and mental health in medical students, and they found that debt per se did not affect students’ performance, unlike their perceptions of their own levels of debt [29]. Using a similar perspective to interpret our results regarding psychological distress, one can conclude that it is related to students’ perceptions of their own financial status, rather than the financial status per se.

### Effects of Syrian war

Described as “the greatest tragedy of the century” by the UN’s high commissioner for refugees [30], Syrian war continues to catastrophically affect various aspects of Syrian people’s lives [25]. The World Health Organization (WHO) estimated strikingly high rates of mental disorders and psychological distress among Syrians [31]. Studies revealed heightened levels of distress, anxiety, fear, frustration, grief, fatigue, and depressed mood in internally displaced Syrians [11].

Poor mental health and high levels of psychological distress were revealed by studies conducted on refugees and displaced people due to the Syrian war [10, 11]. As violence is escalating, people are continuously being displaced. Reports estimate that over half the population of Syria left their homes looking for safer places to live or better living conditions elsewhere, with about 6.8 million of them continued to live in Syria as internally displaced persons [25].

In our study, we keened on highlighting the effects of the Syrian tragedy on medical students’ mental health by asking certain questions that we thought to be relevant and indicatory of being negatively affected by the crisis. The negative effects of the war were prevalent as half of the participants reported being displaced and half of them reported that themselves, or a first-degree family member, had suffered from physical or financial damage due to the war. Nevertheless, analyzing data did not reveal evidence of a significant relationship between those two variables and being psychologically distressed. Indeed, such results do not rule out the role of living in a country riven by war in the detected high levels of psychological distress. Nevertheless, there might be potential uninvestigated war-related factors that contribute to the poor psychological health observed in Syrian medical students as well.

### Limitations

One limitation is the absence of first-year medical students from our sample; a population that was reported to experience substantial stress [1, 23], yielding a potential underestimation of the levels of psychological distress among the overall population of Syrian medical students. In addition, this study used a non-probability, or convenience, sampling method, which may introduce a sampling bias, compromising the generalizability of the results. A response rate could not be computed for the study due to the sampling method used. It would also be hard to verify that our sample is representative of the population of Faculty of Medicine of Damascus University due to the lack of epidemiological data for this population.

Some students who are currently studying at Damascus University are conditionally transferred from their original universities in Syria and were included in our study. In addition, medical students of Damascus University come from different socioeconomic backgrounds, originating from almost all the governorates of Syria. Generalizing the results of this study to medical students of other Syrian universities and to Syria’s general population, however, may be done with caution, considering that governorates of Syria are not affected by the war to a similar extent and that the general population is not subjected to the influence of medical education.

It is worth to mention that DASS can only be used to quantitatively assess psychological disturbance, and not to diagnose clinical psychological disorders [32]. Accordingly, the terms (depression, anxiety, and stress) used in this paper reflect types of psychological distress assessed by the DASS, and not the actual disorders which, indeed, would require a more extensive psychological assessment to be diagnosed.

## Conclusion and recommendations

In summary, we concluded that medical students of Damascus University suffer from high rates of psychological distress.

Strong efforts are required to minimize the burdens of psychological distress in medical students. It was recommended to adjust the curricula in a way that achieves minimal negative effect on medical students’ psychological well-being [33]. We recommend initiating psychosocial and financial support programs that are directed towards Syrian medical students, especially those in early years of study where psychological distress is maximal. Further investigations of causes and consequences of poor mental health in Syrian medical students are essential.

Although efforts are being made to manage the psychological consequences of the Syrian crisis [31, 34], there is still a need for further action. Long-term psychological effects on the general population are well observed in the daily medical practice. Unfortunately, these observations are poorly documented, and many Syrians remain in need for psychological support and care.

## Additional file

Additional file 1: survey of factors associated with psychological distress (an English version). (DOCX 13 kb)

## Abbreviations

AOR: Adjusted Odds Ratio
CI: Confidence Interval
DASS: Depression, Anxiety, and Stress Scale
UN: United Nations
WHO: World Health Organization
