# Supplementary material for: Psychological distress among medical students in conflicts: a cross-sectional study from Syria
Source: BMC Med Educ. 2017 Sep 20;17:173. doi: 10.1186/s12909-017-1012-2 (PMC5607487; doi:10.1186/s12909-017-1012-2)
Supplement: Additional file 1: — survey of factors associated with psychological distress (an English version). (DOCX 13 kb) [file 12909_2017_1012_MOESM1_ESM.docx]

**Additional file 1: survey of factors associated with psychological distress (an English version)**

Please choose one answer for each of the following questions:

1. What is your gender?
   1. Female
   2. Male
2. What is your current year of study:
   1. Second
   2. Third
   3. Fourth
   4. Fifth
   5. Sixth
3. What is your nationality?
   1. Syrian
   2. Palestinian
   3. Other
4. Which statement best describes your marital status?
   1. Single
   2. In a relationship/Engaged
   3. Married
   4. Separated/Divorced/Widowed
5. Which statement best describes your residence status?
   1. Home - with family or relatives
   2. Home – alone
   3. Home - with other students
   4. On-campus housing
6. Did you have to change your residence due to the war?
   1. No
   2. Yes
7. Did you, or any of your 1^st^ degree family members, suffer from a physical or financial damage that was caused by the war?
   1. No
   2. Yes
8. How would you estimate your monthly family income?
   1. Less than 30K S.P.
   2. 30K to 60K S.P.
   3. 60K to 90K S.P.
   4. More than 90K S.P.
9. How would you consider your personal income to be?
   1. Sufficient
   2. Intermediate
   3. Not sufficient
10. Which statement best describes your smoking status (for both cigarettes and waterpipe)?
    1. Not smoker
    2. Current smoker
    3. Former smoker
